# Nutraceuticals in Neurological Disorders

**DOI:** 10.3390/ijms21124424

**Published:** 2020-06-22

**Authors:** Rashita Makkar, Tapan Behl, Simona Bungau, Gokhan Zengin, Vineet Mehta, Arun Kumar, Md. Sahab Uddin, Ghulam Md. Ashraf, Mohamed M. Abdel-Daim, Sandeep Arora, Roxana Oancea

**Affiliations:** 1 Chitkara College of Pharmacy, Chitkara University, Punjab 140401, India; rashitamakker32@gmail.com (R.M.); arundhiman431@gmail.com (A.K.); sandeep.arora@chitkara.edu.in (S.A.); 2 Department of Pharmacy, Faculty of Medicine and Pharmacy, University of Oradea, 410028 Oradea, Romania; 3 Department of Biology, Faculty of Science, Selcuk University Campus, 42130 Konya, Turkey; biyologzengin@gmail.com; 4 Department of Pharmacology, Government College of Pharmacy, Rohru 171207, District Shimla, Himachal Pradesh, India; vineet.mehta20@gmail.com; 5 Department of Pharmacy, Southeast University, Dhaka 1213, Bangladesh; msu-neuropharma@hotmail.com; 6 Pharmakon Neuroscience Research Network, Dhaka 1207, Bangladesh; 7 King Fahd Medical Research Center, King Abdulaziz University, Jeddah 22252, Saudi Arabia; ashraf.gm@gmail.com; 8 Department of Medical Laboratory Technology, Faculty of Applied Medical Sciences, King Abdulaziz University, Jeddah 21589, Saudi Arabia; 9 Department of Pharmacology, Faculty of Veterinary Science, Suez Canal University, Ismailia 41522, Eqypt; abdeldaim.m@vet.suez.edu.eg; 10 “Victor Babes” University of Medicine and Pharmacy, 2 E. Murgu Sq., 300041 Timisoara, Romania; roancea@umft.ro

**Keywords:** neurological disorders, nutraceuticals, herbal therapeutics, food supplements, neurodegeneration

## Abstract

Neurological diseases are one of the major healthcare issues worldwide. Posed lifestyle changes are associated with drastically increased risk of chronic illness and diseases, posing a substantial healthcare and financial burden to society globally. Researchers aim to provide fine treatment for ailing disorders with minimal exposed side effects. In recent decades, several studies on functional foods have been initiated to obtain foods that have fewer side effects and increased therapeutic activity. Hence, an attempt has been made to unravel several extraction techniques to acquire essential bioactive compounds or phytochemicals from therapeutically active food products. This has led to the conception of the term functional foods being meddled with other similar terms like “pharmafoods,” “medifoods”, “vitafoods”, or “medicinal foods”. With a dire need to adhere towards healthy options, the demand of nutraceuticals is widely increasing to combat neurological interventions. An association between food habits and the individual lifestyle with neurodegeneration has been manifested, thereby proposing the role of nutraceuticals as prophylactic treatment for neurological interventions. The current review covers some of the major neurological disorders and nutraceutical therapy in the prevention of disease.

## 1. Introduction

Neurological aliments include a wide array of chronic diseases comprising a highly complicated etiology [1]. A nutrient-deficient diet may lead to disturbances in the central or peripheral nervous system. Globally, more than 10 million people suffer from neurological disorders annually, and this expected to rise in the future. Brain functioning tends to deteriorate with ageing due to neurodegenerative processes, hence leading to the identification of cellular and molecular targets that ultimately leverage better functioning of the brain [2]. About 3.1% of the population in Western countries aged between 70–79 years are considered prone to neurodegenerative diseases while the incidence of disease in individuals of similar age groups in India is 0.7%. The difference is mainly due to varying lifestyle and food habits depending upon the consumption of different ingredients.

Since immemorial times, people have been dependent upon spices and natural products for curing different ailments, which have shown remarkable results [3]. The advancement in science and technology has led to the investigation and utilization of several phytochemicals with therapeutic properties from both plant and non-plant sources, leading to a renaissance in the research of nutrition and human health, thereby creating opportunities for the advancement of novel dietary substances. With this innovation arises a new term called nutraceuticals, which comes from the combination of nutrition and pharmaceutical. The term nutraceutical was coined by Dr. Stephen De Felice in the year 1989 [4]. 

The American Nutraceutical Association defines nutraceuticals as a food or its product possessing health-benefitting properties. They range from dietary nutrient supplements to genetically designed foods, herbal products, beverages, soups, vegetables, fruits, and processed foods, like cereals, etc. [5]. Nutraceuticals are mainly represented by vitamins, minerals, and amino acids, and over 1000 other probiotic compounds have been identified till date. 

The most ancient civilizations that presented evidence of the effective use of food products in medicine and ailing diseases include Indians, a fact even supported by Ayurveda for 5000 years; Chinese; Egyptians; and Sumerians [6]. In brief, a nutraceutical can be defined as a functional food exerting established health benefits apart from its nutritional properties. Evidence reveals that nutraceuticals are emerging as a promising strategy in the management of several chronic diseases, including neurological disorders [7]. The focus of ongoing research in the field of nutraceuticals is the investigation of molecules that are isolated from traditional medicines and how they can be helpful in debilitating and degenerative pathologies [8]. Amidst the potential benefits of nutraceuticals, they still pose certain limitations, such as poor bioavailability, poor brain permeability, metabolism, etc. thereby challenging their beneficial effects [9]. Nutraceuticals in adjunction can strengthen the therapeutic effects of certain medications when used in adjunction by the augmentation of several pathways, such as enhanced re-uptake of inhibited monoamines, thereby providing exceptional neurobiological effects [10]. The current review highlights the potential role of nutraceuticals in brain health and neurological disorders.

Plants served as a treatment source for various diseases through the prehistoric era all over the world. The path to nutraceuticals can be traced historically by establishing links between alternative medicine, including herbalism, apothecary, ethnopharmacology, and phytotherapy [2,11]. The therapy evolved from vegetables, animals, and mineral-sourced compounds and medicinal plants, constituting both instinctive and magical components. Before the concept of nutraceuticals originated, philosophers believed in the concept of diet in the public as well as individual health. From the era of Hippocrates (460–377 BC), i.e., 2000 years back, to the rising phase of modern medicine, it was recognized that the difference in diseases depends upon the food consumed in society [12]. 

In the year 1989, the New York’s Foundation for Innovation in Medicine, an educational foundation, came up with term “nutraceuticals” to promote research rapidly in the biomedical sector [13]. The Europeans acquired traditional knowledge from Asian countries, and benefited the most [14,15]. The rational use of medicines urged the role of pharmacists and the discovery of principles in drug action with the simultaneous development of modern drug development with clinical trials. The Indian history, including the Unani, Ayurveda (including Sushruta, Samhita, and Charaka), Ashtavaidya, and Siddha system of medicines, is renowned for possessing the art of healing procedures [16,17].

There is an abundance of unexplored food products and nutrients that possess valuable biological activities. At present, the nutraceutical industry is the rapidly establishing segment of today’s food market [18], with a 30 billion US dollars market growing annually at a rate of 5% per annum [19]. The current stand of nutraceuticals and knowledge accumulated about it poses a great challenge for nutritionists, food technologists, physicians, and food chemists [20]. 

In the process of pharmaceutical development, clinical testing on animals and humans is a must and the results obtained verify the therapeutic effects of the drug [21,22,23]. While no established methods for verification of the therapeutic effects of nutritional foods were indicated in the past, in the recent times, it has been scientifically proven that food compounds can prevent lifestyle-related disorders. Nutraceuticals offer several advantages, including an increased significance of a healthy diet and aiding a longer life. Apart from its beneficial effects in medical conditions, it also assists proven psychological benefits; hence, they are the most popular in preventing neurological disease conditions [24,25]. Due to the lesser perceived side effects, more populations, mainly elderly, tend to adhere more towards nutrient-rich foods for lifestyle-related disorders. 

The aims and scope of the manuscript is to raise the awareness of the readers about the use of nutraceuticals in the management of neurodegenerative and psychotic disorders through the use of ingredients that are easily available and tend to show proven neuroprotective effects. The current review highlights the potential role of nutraceuticals in brain health and neurological disorders.

## 2. Methodology

Before commencing the review article, a deep literature survey on nutraceuticals in neurological disorders was performed. Research and review articles from various search engines and scientific databases, such as Pubmed, Medline, Science Direct, Google, Scopus, Cochrane library, etc., were assessed and thoroughly read for a deep understanding of the topic and to evaluate the currently employed psychoactive and neuroprotective nutraceuticals. After the literature survey, the article writing was initiated. The total time span for the completion of the review article was approximately 2 months.

## 3. Nutraceuticals and Its Categories

Nutraceuticals fall under nonspecific biological therapies and are used in the prevention of symptoms of mild disorders to highly toxic malignancy. Their role as a neuroprotective is well pronounced and highly acknowledged. They can be categorized considering the following criteria:

### 3.1. Food-Based Nutraceuticals or Traditional Nutraceuticals

This category includes food products obtained directly from nature without any change in their original constituent form. These include fruits, vegetables, grains, meat, fish, eggs, and dairy that provide several benefits beyond basic nutrition [26,27]. 

#### 3.1.1. Nutrients

The primary metabolites of substances like minerals, fatty acids, vitamins, and amino acids possess well-established nutritional properties in the metabolic pathways. These nutrients in combination with animal and plant products have several benefits in curing neurological disorders. The planting of nutrients can be used in preventing brittle bones, uplifting hemoglobin, and strengthening muscle power and neuronal transmission. Fatty acids and its compounds enhance brain functioning and aid a decrease in cholesterol present in the arteries, tending to show its hypolipidemic effects [4,5].

#### 3.1.2. Herbals or Extracts and Concentrates of Botanical Products

The combination of nutrients and herbals poses an excellent impact on lifestyle-related disorders, including mental health [28,29]. Tannin-containing compounds, such as lavender, help in releasing stress and lowering blood pressure. Flavonoids have been clinically proven to prevent diabetes, cardiovascular disorders, and kidney abnormalities based on their antioxidant potential, containing compounds, such as psoralen, which is obtained from parsley and also possess carminative and diuretic properties. Terpenoid-containing compounds, such as peppermint and menthol, are used in respiratory conditions. Many other commonly used herbs, such as aloe vera, possesses anti-inflammatory and dilating properties, hence it is used in wound healing; ephedra possess bronchodilator and vasoconstriction effects, hence it is used for bronchospasms [1]. The most commonly used food ingredients, garlic and ginger, possess anti-inflammatory and chemotherapeutic properties, are used in hypertension, and are a strong immunity booster [30,31,32]. Not only herbal products but also the phytoconstituents they possess can also be categorized under nutraceuticals, for example, vegetables contain carotenoids, which boost immunity, mainly killer cells, and possess anticarcinogenic properties [33]. Non-carotenoid foods, such as chickpeas and soya beans, aid in the removal of cholesterol. Curcumin obtained from turmeric, one of the most common ingredients in the kitchen, can be classified under phenolic acids and possesses the highest antioxidant activity and acts as an anti-inflammatory. Dietary supplements, mainly antioxidant-rich foods, such as green tea, ginger, cumin, etc., have shown promising effects in weight loss [34]. They have also been studied for their efficacy in neurological interventions, such as depression [35]. They also include enzymes and glandular extracts, and can be consumed in all dosage forms, including capsules, powders, tablets, etc. 

#### 3.1.3. Probiotic Microorganisms

The term probiotic was coined by the famous scientist Metchnikoff. They are highly advantageous in relation to gastric and intestinal physiology. They possess antibiotic properties and aid in the removal of toxic flora from the gut. A healthy diet leads to a healthy brain and body.

The consumption of probiotics has been a breakthrough in the management of gastrointestinal disorders. After these results, probiotics have also been initiated for their consumption as supplements in the form of capsules and probiotic beverages. Thus, modern day probiotics claim to be effective in all health conditions from diarrhea to neurological conditions, such as depression and Alzheimer’s, and are challenged for their therapeutic effects. There is a great need to explore probiotics, as published research on their safety is lacking. It is difficult to differentiate the benefits of probiotics from its contraindications. In instances with high risk of infection in patients with compromised immune system, the probiotics may be moderately effective therapeutically [36,37]. 

#### 3.1.4. Nutraceutical Enzymes

Enzymes or biocatalysts are protein structures and are synthesized by cells. They cause metabolic processes to occur faster and are mainly beneficial in medical problems related to the gastrointestinal tract, such as gastroesophageal reflux disease, constipation, diarrhea, etc. Enzyme supplements provide the least advantages in neurological health, but recently, some therapies have been procured to cure rare disorders, such as Hunter syndrome, Gaucher disease, etc. They are highly economical as they are obtained from both plants and animal sources.

A large number of advantages are offered upon the consumption of food-based nutraceuticals. Nutraceuticals obtained from foods, such as garlic, ginger, turmeric, dairy products, carotenoids, etc., are much heathier and can provide all the essential nutrients required by our body. They are easily available in grocery stores and prevent the exacerbation of severe life-related disorders, such as diabetes, and even cancers. Having good mental health is a priority, and a good diet can be the most appealing option for neuroprotection. However, they pose certain disadvantages too. The most stressed drawback of food-based nutraceuticals is their safety. There is still a dire need to explore functional foods for their safety before they are released in the market for consumption in raw forms. All substances are poison unless consumed in a finite amount. It is evident that a food that is highly active as an anticarcinogen can simultaneously act as a cardiotoxic. Thus, administration of the desired dose is recommended [30,31,32,38]. 

### 3.2. Non-Traditional Nutraceuticals

These include foods obtained from the breeding of agricultural products and nutrients, such as orange juice fortified with calcium, vitamins and minerals in cereals, etc. Cultural scientists have successfully invented techniques and have changed the nutritional content of crops, and more research is being carried out to improve the quality of nutrition in crops [39,40].

#### 3.2.1. Fortified Nutraceuticals

These are the type of nutraceuticals that are designed from breeding at the agricultural level by enhancing nutrients, such as minerals in cereals, increasing calcium, folic acid, iron in flour, making milk fortified with cholecalciferol for the treatment of vitamin D deficiency, etc. [41,42]. 

#### 3.2.2. Recombinant Nutraceuticals

Nutraceuticals obtained from the application of biotechnology in food products are called recombinant nutraceuticals. These nutraceuticals are amongst the most commonly used category, including the extraction of nutrients from certain food products, like dairy products, cheese, and bread, to extract the enzyme that is therapeutically beneficial if used at optimum levels [2,18].

Non-traditional nutraceuticals have emerged to boost a larger extract of nutrients in already existing food supplements and aim to provide more benefits in the same volume of food consumption. It offers several advantages and is a blessing in the era of sedentary lifestyles, yet it poses certain threats. The production of nutraceuticals still lacks regulations by the Food and Drug Administration (FDA) [43]. There are many companies that synthesize non-traditional nutraceuticals of poor quality to obtain larger profits and margins. Additionally, the bioavailability of enhanced nutrients is not monitored and is generally poor in various scenarios. The testing of nutraceuticals is not as controlled as pharmaceuticals, clearly indicating no defined regulations.

### 3.3. Based on the Mechanism of Action 

Based on the therapeutic properties possessed, nutraceuticals have been further classified into antibacterial, antifungal, antioxidants, anti-inflammatory, and antiobesity categories to distinguish their role and assess their uses. Food-borne infections are the cause of a wide number of deaths due to infection. There are many bioactive compounds that have been used as a potent antibacterial therapy in the management of infectious disorders, such as carsonic acid (terpenoids), quercetin (polyphenols), etc. They are obtained from a wide number of fruits and vegetables [44]. Traditional foods have been consumed for many years for their therapeutic activity as an anticancer, antidiabetic, etc. Various studies have led to the formation of medicinal mushrooms as a good candidate for antifungal therapy with the least side effects. These mushrooms are widely popular all over Asia [45]. Nutraceuticals have been used in various inflammatory disorders, including rheumatoid arthritis, because of its anti-inflammatory action [46]. Foods rich in antioxidants, carotenoids, and tocopherols possess higher anti-inflammatory activity. Nutraceuticals are renowned for their free radical scavenging properties and antioxidant action. This antioxidant activity leads to its role in the management of other related disorders, such as obesity [47]. 

As discussed above, consuming anything in excess acts as a poison. The consumption of nutraceuticals based on their mechanism of action is highly supportive. Consuming any compound for its therapeutic activity tends to decrease the chances of evident toxic traits associated with it and benefits the person for the desired diseased condition. Additionally, it leads to monitored administration of nutraceuticals and prevents any toxic effects associated with high doses.

### 3.4. Based on the Chemical Nature of the Products

**Figure 1:**
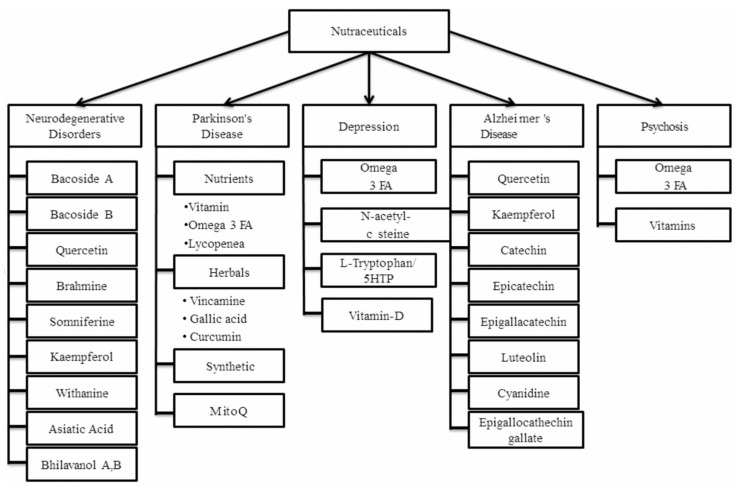
Some of the most commonly used nutraceuticals that can be employed as adjunctive therapy in the management of neurological degeneration, Parkinson’s disease, depression, Alzheimer’s disease, and psychosis simultaneously [1,2,4].

Nutraceuticals can also be classified based on the secondary metabolites they possess, such as fatty acids, carbohydrates, and amino acid-based compounds, as the origin for every nutraceutical is different depending on the natural source [48]. Most commonly used nutraceuticals as adjunctive therapy in different neurological disorders are classified as it is presented in Figure 1.

The chemical nature of the compound defines the activity it is associated with. Consuming nutraceuticals based on their chemical nature can enhance the therapeutic activity and minimize the risks of toxic actions that can be experienced. However, the results are not as expected. The functioning of every individual is different, and some active compounds have a tendency to react with host molecules and produce toxic traits.

## 4. Nutraceuticals in Ameliorating Neurodegeneration

Neurodegenerative disorders mainly develop by protein misfolding. Abnormal misfolding of the proteins τau and amyloid-β (Aβ) leads to the progression of Alzheimer’s disease; traumatic brain injury can be induced by modifying τau, trans active response d(eoxyribo)n(ucleic) a(cid) (TAR DNA) -binding protein-43 (TDP-43), and Aβ proteins; while τau and TDP-43 misfunctioning can subsequently induce epilepsy and various other tauopathies. The cytotoxic cascade of molecular and cellular events is mainly induced by protein Aβ in Down syndrome, and α-synuclein in Parkinson’s disease, leading to detrimental consequences and further degeneration [49,50,51,52]. These misfolded proteins further stimulate nuclear factor kappa-light-chain-enhancer of activated B cells (NF-κB ) activation, which causes the production of inflammatory cytokines (such as tumor necrosis factor-α (TNF-α), interleukins-1β (IL-1β), etc.), and leading to the activation of destructive molecules (like cyclooxygenase (COX-2), inducible nitric oxide synthase (iNOS)); the actions mentioned are the results of reactive oxygen species (ROS) release and glutamate-induced oxidative damage, causing the dysfunction of mitochondria and toxicity [19,53,54]. Additionally, the misfolded proteins further dysregulate the signaling of GSK3β with simultaneously provoked inflammatory cytokines, which leads to hyperphosphorylation of tau proteins, and causes an increased synthesis of cholesterol. Furthermore, it also results in the formation of lipid raft, harboring misprocessing and misfolding of proteins due to the promotion of enzymes, thereby setting up a vicious cycle. Moreover, misfolded proteins dysregulate various signaling pathways – such as extracellular signal regulated kinase (ERK), cyclic adenosine monophosphate (cAMP) response-element binding signaling (CREB), and protein kinase A/protein kinase B (PKB/PKA), and cholinergic functions, leading to defects in cognitive functions and degradation of the synaptic process [55,56,57]. Nutraceuticals can tend to modify the cellular and molecular cascade and can lead to the prevention of neurodegeneration by targeting proteins that are misfolded practically at all levels and act as supplementation therapy. 

**Figure 2:**
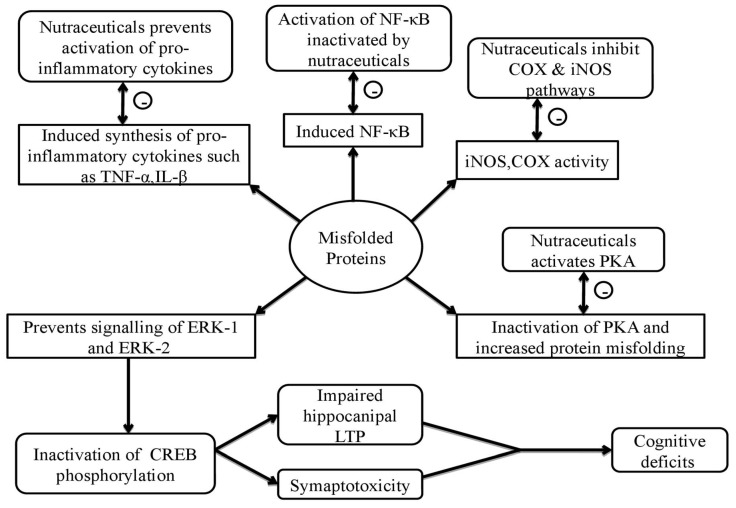
Summarized pathogenesis of misfolded proteins and neurodegeneration mediated upon their activation. The misfolded proteins lead to the activation of cascade of inflammatory proteins, such as nuclear factor kappa-light-chain-enhancer of activated B cells (NF-κB), inducible nitric oxide synthase (iNOS), and cyclooxygenase (COX), and activation of interleukins and inflammatory cytokines, which leads to inflammation and further neurodegeneration. Inhibition of these cascade proteins by active nutraceuticals tends to provide neuroprotective action.

It has been found that nutraceuticals have antioxidant, anti-hypercholesterolemia, and anti-inflammatory effects with simultaneous production of the enhanced cholinergic system due to acetylcholinesterase inhibition [58]. Nutraceuticals, when used for their therapeutic potential, can easily replace synthetic drug ingredients, such as donepezil, tacrine, rivastigmine, and galantamine, which act by inhibiting acetylcholinesterase enzyme; statins like rosuvastatin and atorvastatin, which act by inhibiting 3-hydroxy-3-methylglutaryl coenzyme A (HMG-CoA) reductase; alpha tocopherol or vitamin E; aspirin, ibuprofen, and other cyclooxygenase (COX) inhibitors under the category of non-steroidal anti-inflammatory drugs (NSAIDs); etc., as these compounds possess evident side effects. Hence, nutraceuticals offer an all-in-one effective alternative in the management of neurological disorders due to their affordable prices and availability and decreased side effects [59,60]. The pathogenesis of misfolded proteins that mediate neurodegeneration is briefly represented in Figure 2.

The main nutraceuticals in neurological disorders include bacoside A, bacoside B, and brahmine (as they were classified in Figure 1). Bacoside A and bacoside B are saponin derivatives, while brahmine is an alkaloid derivative, which is obtained from brahmi (*Bacopa monnieri*) [61]. It is a renowned nootropic plant, which has been used in Ayurveda for its neurocognitive-enhancing properties. The human brain is highly susceptible to neurodegeneration due to an increase in oxidative stress and the generation of free radicals due to a high metabolic rate; poor antioxidant activity of catalase, glutathione peroxidase, and other free radical scavenging enzymes; and the presence of unsaturated fatty acids in the membranes of cells [29].

The plant is a proven antioxidant. Through various studies it has been established that the protein amino group side chains, after the reaction with d-galactose, lead to the generation of amadori products that result in advanced glycation end products (AEGs) [62]. The glycated products lead to a 50-fold increased production of free radicals than non-glycated products, ensuring oxidative stress. Administration of phytoconstituents, mainly bacosides A and B and brahmine, significantly decreased the number of AEGs and prevented aluminum-mediated neurotoxicity in the cerebral cortex region of the brain and is effective in the prevention of neurodegeneration [19].

### 4.1. Quercetin and Kaempferol

The generation of free radicals in the brain leads to inhibition of amyloid β1-42 proteins and their aggregation and also leads to fibril destabilization. Quercetin and kaempferol have been proven to decrease the levels of free radicals remarkably [63]. They also prevent the activation of NF-kB, which further prevents the activation of proinflammatory cytokines, mainly interleukins. It is among the most commonly explored phytoconstituents, mainly obtained from the leaf extract of *Gingko biloba*, in the prevention and cure of cognitive disorders [64]. They are also highly effective in improving the circulation of blood in the brain and prevent the progression of Alzheimer’s disease [65]. 

### 4.2. Withanine

Withanine is the chief steroidal alkaloid obtained from ashwagandha, also known as Indian ginseng [66], which has been used for its memory-boosting and neurocognitive-enhancing properties for more than 2500 years. It possesses high antioxidant potential and can be used to improve oxidative stress-mediated neurodegeneration. The methanolic extract of ashwagandha root exhibits memory-boosting action and inhibits the enzyme acetylcholinesterase, which is of great significance in neurodegeneration as it indirectly facilitates the transmission of cholinergic neurons and is highly recommended in the treatment and management of Alzheimer’s disease [19]. The levels of catecholamines, including serotonin, are also augmented besides the antioxidant activity by maintaining the levels of antioxidant enzymes, mainly glutathione and catalase. Withanine inhibits the activation of nitric oxide, which further reverses oxidative stress, and presents remarkable neuroprotective effects. Somniferine, also obtained from ashwagandha, is also widely used for its neuroprotection and memory-enhancing effects. 

### 4.3. Asiatic Acid

Gotu kola has been used for its memory-enhancing properties in Ayurveda and also aids in improving learning. Its principal phytoconstituent, namely asiatic acid, is chiefly responsible for the neuroprotective actions. It acts by decreasing the levels of malondialdehyde while simultaneous increasing glutathione. Malondialdehyde is a by-product formed post peroxidation of lipids which acts as an utmost important marker for the detection of free radicals of oxidative stress-mediated neurodegeneration. Asiatic acid increases the levels of free radical scavenging enzymes, such as glutathione, and augments its antioxidant medication protection of neurodegeneration [64].

### 4.4. Bhilavanol A and Bhilavanol B

Bhilavanol A and bhilavanol B, which are chiefly obtained from bhallaatak, inhibit the activation of acetylcholinesterase and are highly effective against stress-mediated neurodegeneration. Ingredients of the Mediterranean diet, such as coffee, extra virgin olive oil, walnuts, etc., also improve memory and are highly beneficial. The phenolic compounds extracted from plants are highly emphasized as they possess maximum therapeutic benefits [64].

## 5. Nutraceuticals in Alzheimer’s Disease (AD)

Alzheimer’s disease (AD), also known as senile dementia of the Alzheimer type (SDAT) or the primary degenerative dementia of the Alzheimer’s type (PDDAT), is the most common form of memory loss (Linseman, 2009). Pronounced nutraceuticals that are helpful in the management of AD include super essential antioxidants, which can be employed in the treatment of all chronic diseases due to oxidative stress, which exhibits a crucial part in neurological disorders, including AD [67]. The process of ageing and lack of intake of dietary antioxidants accelerates oxidative stress, causing disease progression and stimulation. Various studies have reported an association between the intake of higher amounts of dietary antioxidants and diminished risk in patients with AD, which is highly imperative as disease prevention is considerably cooler than treating it [68]. Additionally, researchers suggest that the prevention of AD is not as complex as assumed. The consumption of food products that are rich in polyunsaturated fatty acids and saturated and trans fatty acids tends to suppress neurodegeneration while foods rich in trans-fat can enhance neurodegeneration [28]. The use of antioxidants for treatment is a hopeful option for slowing the progression and advancement of diseases [69]. Some of the compounds beneficial in AD are described in the Section 5.1, Section 5.2, Section 5.3, Section 5.4 and Section 5.5.

### 5.1. Flavonoids

The main employed flavonoids in neurogenerative disorders, mainly Alzheimer’s, include catechin, epicatechin, epigallocatechin, and epigallocatechin gallate. These are a group of commonly found polyphenolic compounds mainly extracted from the human diet. The main resources of flavonoids include fruits, vegetables, and drinks, such as wine, tea, and cocoa. Flavonoids and their metabolic products possess neurological-modulating actions and have been studied to interact with the neuronal-glial signaling pathway, which is mainly involved in the survival and functioning of neurons [70]. The cerebral flow of blood is also modulated by upregulated activity of antioxidant proteins and enzymes, which causes synaptic plasticity and repair of neuronal functions by inhibiting the process of neuropathology in the brain mainly associated with AD [71].

### 5.2. Carotenoids

About 700 diverse members of the carotenoid family have been identified to date, 40 of which are found in human tissues and blood. The major carotenoids present in humans include lutein, zeaxanthin, lycopene, and β-cryptoxanthin, including α and β carotenes. The antioxidant activity of carotenoids can be identified on the basis of their chemical structure setting. They are fat-soluble pigments and can mainly be extracted from fruits and vegetables that are orange, deep-yellow, and red in color [63]. Astaxanthin, a seafood-derived carotenoid, has been extensively studied for its anti-inflammatory and antioxidant potential in in vivo and in vitro animal models, and its microcirculatory protective functions and mitochondrial protective functions have been identified, suggesting it is a potent neuroprotective compound. Patients with severe or moderate AD lack major carotenoids, such as lutein and beta carotene, compared with patients with mild AD [72].

### 5.3. Crocin

Crocin is a chief phytoconstituent obtained from saffron (*Crocus sativus*). It has been used for ages for its antispasmodic, neurine sedative, gingival sedative, expectorant, stimulant, and carminative properties. Saffron has been proven to act in the prevention of epilepsy, depression, and inflammatory disorders. Crocin is also known to improve learning and enhance memory based on its long-term potential being blocked by ethanol, and hence, it is used in neurodegenerative disorders, such as AD. Crocin tends to improve cognition by ADAS-Cog and CDR-SD-mediated enzymes in patients with mild to moderate AD. Through various studies it has been concluded that crocin can significantly alter the levels of oxidative markers in the region of the hippocampus and abolish the deleterious effects on learning and memory due to chronic stress [73].

### 5.4. Cyanidin

The other major compounds include cyanidin (anthocyanidins), which is mainly obtained from cranberries, strawberries, etc., and exert potent anti-inflammatory and neuroprotective action by suppressing the activation of proinflammatory cytokines and ultimately brain cell damage. The main role can be attributed to the inhibition of phospholipase A2, which is chiefly involved in the signaling of proinflammatory cytokines and oxidative stress parameters, the inhibition of which presents remarkable neuroprotection. 

### 5.5. Luteolin

Luteolin and apigenin are flavones, which possess remarkable neuroprotective activity. The principal sources of these flavone-containing compounds include rosemary, parsley, and celery. These phytoconstituents possess remarkable pharmacological benefits, mainly the ability to protect DNA against hydrogen peroxide-mediated toxicity, further preventing inflammation and cell damage in Alzheimer’s [74,75].

## 6. Nutraceuticals in Parkinson’s Disease

Parkinson’s disease (PD) is a neurological disease with impaired dopaminergic neurons in the substantia nigra par compacta region of the brain, leading to drastic depletion of dopamine (DA). Factors, such as oxidative stress, depletion of antioxidants, damage to mitochondria, etc., contribute to neurodegeneration leading to PD [76]. Anti-Parkinson’s diseases provide symptomatic relief by supplementing dopamine and preventing symptoms of motor abnormalities and gait, and providing neuroprotection [77]. Therefore, a wide range of drug molecules are implemented, which act by the activation of several pathways of the prevalent pharmacotherapy. Abundant studies on vitamins and their supplementation in animals and clinical studies have been performed, which depicted mixed outcomes in managing the symptoms of PD; therefore, there is a need for more research and established evidence on their effects on PD. 

Several vitamins, including vitamin B3, vitamin B9 or folate, vitamin B12, vitamin B6, vitamin D, vitamin E, and vitamin C, can be used in Parkinson’s disease [78]. The anti-Parkinson drugs currently employed prevent disease progression by providing symptomatic relief only. The main challenge lies in recognizing the ideal lead molecule, which, besides targeting multiple pathways and curing disease, is also least toxic to humans. With this as the principal, a wide number of herbal and natural products have been studied clinically for use in PD [79] to evaluate and clarify if such herbal molecules can be implemented as an independent or adjunctive therapy in disease management [80]. It is tough to retrospectively study the effect of a herbal drug, food product, or supplement in a large population due to the high levels of variance and unreliability of results based on patients’ statements and contributing lifestyle patterns. Thus, these challenges during clinical trials on synthesized herbal products restrict the emergence of identified lead molecule in the market [81]. Natural products exert favorable effects in PD by blunting the different pathologic pathways inducing it, like oxidative stress, dysfunction of mitochondria, neuroinflammation, and apoptosis.

### 6.1. Targeting the Dysfunction of Mitochondria and Oxidative Stress 

Uninhibited oxidative stress and free radicals in association with the dysfunction of mitochondria leads to compromised cellular metabolism and energy homeostasis, thereby impacting the functioning of the brain, and leading to neurodegenerative disorders, including PD. However, it is not clear if the dysfunction of mitochondria is a consequence or cause of neurodegeneration. The mutations in mitochondrial DNA in dopaminergic neurons and defective chains in the respiratory system in patients with PD have been hypothesized as the mechanism that induces mitochondrial dysfunction. The mitochondria in cells regulate the supply of ATP and calcium to release stored neurotransmitters into the synaptic cleft and depolarizing neurons, hence protecting cells by fission and fusion. The role of α-Syn was demonstrated in the morphological maintenance of mitochondria and enhanced efficiency of ATP synthase. The aggregates of α-Syn lead to compromised functioning of bioenergetic mitochondria and upregulate the generation of reactive oxygen species, which causes an unbalance between the oxidative status and death of primary neurons in rats. 

Neuromelanin (Nm), a crucial pigment present in dopaminergic neurons, is highly protective against oxidative stress. Nm can easily chelate multiple ions, including iron and zinc, to maintain balance in the redox system. Surplus iron concentrations have a significant role in the pathology of PD as abundant iron stores and Nm levels can aggravate neurotoxic events, which triggers autooxidation of DA and leads to neuroinflammation. The components of food besides nutraceuticals have been successfully shown to prevent or delay the progression of disease by preserving the functioning of mitochondria, further strengthening its role as a major pathological mechanism in PD [82,83]. There are several nutrients, phytochemicals, or synthetic compounds that can act and prevent disease progression by preventing mitochondrial dysfunction. 

Amongst nutritional supplementation, coenzyme Q10 (CoQ10) and fish oil can be used efficiently in PD management as they are the key components of the electron transport chain and are actively involved in the production of ATP, counteracting 1-methyl-4-phenyl-1,2,3,6-tetrahydropyridine (MPTP)-mediated neurotoxicity and blocking the transfer of electrons between complex 1 and other complexes. Apart from this, polyphenols also possess multidimensional features to counteract the pathology of PD as they can easily surpass the blood–brain barrier and present favorable actions by improving motor and gait abnormalities in patients by protecting dopaminergic neurons and limiting free radicals. Lycopene, as initially studied, is a lipid-soluble acyclic carotenoid obtained from red-colored fruits and vegetables, mainly tomatoes, which exerts an antioxidant effect and has presented neuroprotective action in a study conducted on 1-methyl-4-phenyl-1,2,3,6-tetrahydropyridine) (MPTP)-induced mice, and has been shown to enhance the levels of dopamine (DA) in the striatum region. The therapeutic effects of lycopene are dedicated to its antioxidant activity accompanied with neurobehavioral deficits and an increase in the activity of superoxide dismutase (SOD) and nicotinamide adenine dinucleotide (reduced form) (NADH) dehydrogenase at the striatal level besides increased glutathione and decreased malonaldehyde concentrations. 

Fish oil is highly rich in omega-3 fatty acids, such as eicosapentaenoic and docosahexaenoic acids, thereby showing neuroprotective effects by multiple pathways. EGCG or epigallocatechin-3 gallate is one of the most prevalent polyphenols obtained from *Camellia sinensis* and has successfully shown neuroprotective activities due to its ability to surpass the blood–brain barrier (BBB). The catechol-like structure of EGCG is responsible for the radical scavenging activity and iron chelation property of the phytoconstituent. It substantially improved motor functions in diseased patients and decreased neurotoxicity by enhancing DA levels in the striatal region of the brain. Ginseng and its derivatives, ginsenosides, demonstrated neuroprotective activity in several studies on PD. The antioxidant activity of ginsenoside is related to its ability to manage the levels of glutathione and the reactive oxygen species-mediated NF-kB pathway, and regulation of the transport of iron and related proteins, thereby causing depleted stores of iron in the nigral region of the brain. Vincamine, an alkaloid obtained from the vinca plant, has proven anti-PD activity via different mechanisms of action. It possesses vasodilation activity and causes muscle relaxation of the capillaries in neurons, causing an increased flow of nutrients and glucose to the brain with a parallel increase in ATP generation through the Krebs cycle. 

Oxidative stress and iron are also targeted by vincamine to improve the production of DA and lessen the neuronal damage produced. Hence, the role of vincamine and its derivatives, vinpocetine, can be summarized in the management of PD by reducing the synthesis of ROS and iron-chelating molecules. Another synthetic compound, namely mito Q, is also used in the management of PD. The structure of mito Q comprises a lipophilic cation called triphenylphosphine, which is the chief constituent responsible for its antioxidant activity and maintains the functioning of the respiratory chain. A natural antioxidant compound named apocynin is being investigated for its PD-protective activity [81,82].

### 6.2. Endoplasmic Reticulum (ER) Stress Pathway and Protein Misfolding and Aggregation

Abnormally misfolded proteins evoke stress in the ER and lead to unfolded protein responses (UPRs), which further cause ER-mediated aggregation and degradation of proteins and autophagy. The principal aim of therapies that act by targeting this mechanism is to prevent the aggregation of proteins and formation of misfolded proteins. The inability to clear aggregated proteins or remove damaged organelles can cause apoptosis or cell death and lead to neurodegeneration. Vitamins are the most commonly used nutrients in patients with PD. However, hydrophobic antioxidants, such as vitamin A, beta carotene, and CoQ10, also possess anti-fibrillogenic properties. Vitamin A promptly inhibits the deposition of intracellular α-Syn in vivo. Crocin is another phytoconstituent that possesses neuroprotective properties in several central nervous system (CNS ) disorders, which can be ventured through successive results obtained from in vivo and in vitro studies. This carotenoid decreases the expression of CHOP and binding immunoglobulin protein (BIP)/Grp78 and inhibits the activation of various factors responsible for apoptosis, including proapoptotic factor caspase 12 PC12 cells, after exposure to MPP. 

Bicalein is a flavonoid isolated from the roots of *Scutellaria baicalensis georgi*, a plant obtained from Iran. This compound significantly prevents fibrillation and neurotoxicity by pausing the formation of oligomer of α-Syn. This flavone tends to induce autophagy, decrease inflammation and inflammatory cytokines, and inhibit apoptosis, thereby restoring the levels of DA in an MPP-induced model in mice. Resveratrol represents a potent pharmaceutical compound due to its solubility and stability [84]. It increases metabolic turnover and enhances the microflora in the gut but possesses low BBB permeability, hence it can compromise the bioavailability of polyphenol compounds in the brain [82]. 

**Figure 3:**
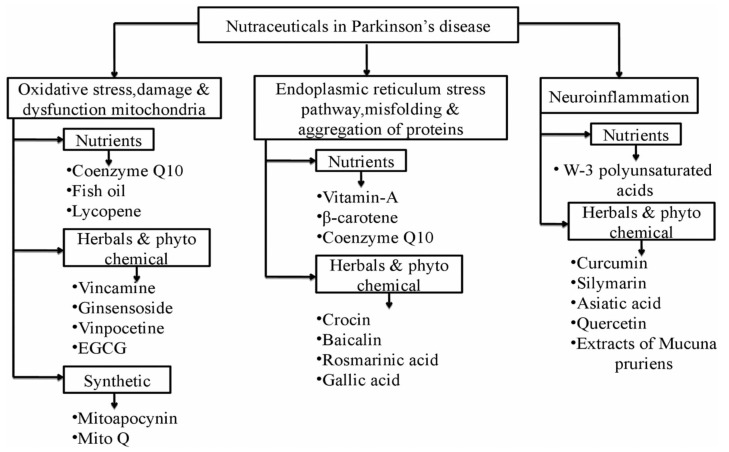
Nutraceuticals in Parkinson’s disease act by three pathways. 1. By preventing oxidative stress, which leads to protection of mitochondria from further damage and dysfunction and ultimately maintains energy homeostasis and cellular metabolism; 2. Activation of misfolded proteins and their aggregation induces stress in endoplasmic reticulum (ER), which further causes autophagy and degradation of neuronal proteins. 3. Inflammation in neuronal cells is the main cause of neurodegeneration and onset of Parkinson’s disease [77,78,79,80].

Nutraceuticals having action in Parkinson’s disease are summarized in Figure 3.

## 7. Nutraceuticals in Depression

Depression is a mental disorder, which is mainly characterized by a sad or depressed mood combined with a decreased interest in any social activity, leading to an impaired routine. Its prevalence is about 15% with an annual incidence of 7%. It poses a huge burden on society with an increased cost of life quality as a depressed person is less productive and is at a higher mortality risk. Omega-3 fatty acids and folic acid have generally been effective for unipolar depression, particularly as an adjunctive therapy, with increasing evidence for its efficacy as a monotherapy. The nutrients obtained from dietary products are critical for proper brain functioning as a relationship between the quality of food and brain health and mood has been identified and studied [83], leading to the application of nutraceuticals as supplements. 

A whole-grain diet rich in nutrients, such as zinc, folic acid, omega-3 fatty acids, and several other essential macro and micronutrients, can trigger the functioning of the brain and have evidently shown results in the management of depression [10]. 

The mechanisms of action of some nutraceuticals in depression are presented in Table 1.

Apart from the nutraceuticals mentioned above, *Hypericum perforatum*, commonly known as St. John’s Wort, has also been studied for its remarkable antidepressant activity. The plant is a highly rich source of flavanol glycosides, including major components, such as rutin, quercetin, hypericin, and hyperforin. The plant acts as an antidepressant by inhibiting the enzyme monoamine oxidase (MAO). Carbon dioxide (CO_2_) extract enriched with hyperforin and adhyperforin inhibited the re-uptake of neurotransmitters, such as norepinephrine, serotonin, and dopamine, and showed antidepressant effects [87].

## 8. Nutraceuticals in Psychotic Disorders

Nutraceuticals, besides the functional roles studied, also play a key role in the management of mood disorders and psychotic disorders, such as schizophrenia and bipolar disorder [88]. They are mainly employed as an adjunctive therapy and sometimes as a monotherapy in patients who are in dire need of psychotic care. Nutraceuticals strongly amplify the therapeutic efficacy of the medications employed by strengthening the neuroprotection by enhancing the inhibited re-uptake of monoamines and showing neurobiological effects [89,90,91,92], thereby improving the efficacy of psychiatric medicines and ameliorating their side effects. 

The commonly used nutraceuticals in psychosis include omega-3 fatty acids and vitamins. There are two main types of polyunsaturated fatty acids in the human body: Those of the omega-6 series, such as arachidonic acid (AA), obtained from linoleic acid, and those of the omega-3 series, obtained as alpha-linolenic acid. The latter include eicosapentaenoic acid (EPA) and docosahexaenoic acid (DHA) [93]. All of them are important components of the phospholipid cell membrane and are essential for survival of the human body. However, as the body cannot synthesize them, they must be obtained through the diet. On the molecular level, omega-3 EPA and DHA have properties that are of interest in psychotic disorders. They improve dopaminergic and serotoninergic neurotransmission. They decrease microinflammatory and oxidative stress. They modulate the functioning of mitochondria, which are the main source of oxidative stress [94]. Additionally, they protect against toxicity due to apoptosis and regulate gene expression of brain-derived neurotrophic factor (BDNF). 

Vitamins are organic compounds that the human body cannot synthesize in adequate amounts, so they need to be obtained through the diet. The efficacy of interventions with vitamins in schizophrenia has been reviewed recently. 

Table 2 summarizes the most relevant information regarding the commonly used nutraceuticals in various neurological aliments.

## 9. Conclusions

Nature has provided us with valuable herbal molecules with high potential in the cure and prevention of life-threatening diseases and lifestyle-related disorders, including neurodegeneration. The role played by phytonutrients in dealing with neurodegeneration and preventing cognition has been evidently described in various studies. The curative effects of nutraceuticals can be attributed to their neuroprotective, anti-inflammatory, antioxidant, hypolipidemic, and healing properties, which target different ligands and receptors to enhance protein synthesis, which ultimately leads to neuroprotection. The folding of proteins and their degradation can be inhibited, leading to a healthy nervous system. The experimental research on plant products has provided new directions for the affordable treatment of neurodegenerative diseases in this era of many public health system crises.

A changing lifestyle has deteriorated the body’s defense mechanism to scavenge free oxygen radicals by suppressing antioxidants, resulting in overloaded oxidative stress. Increasing age also tends to decrease levels of antioxidants in our body, thus attracting chronic illnesses in humans. Therefore, for years, the focus has been placed on targeting a variety of nutraceuticals for their therapeutic properties. Products containing antioxidants, such as vitamins, intrinsically act by scavenging free radicals and stimulating the synthesis of antioxidants in the body. The current review highlights the merits and demerits of nutraceutical therapy and its susceptibility to preventing disease progression in neurological disorders. Though nutraceuticals have been shown to exhibit remarkable properties, the response varies from person to person. Consuming them in acceptable and recommended dosages promotes good neurological health and keeps diseases at bay; hence, they are the best options for curing lifestyle-related mental disorders, like depression.

## Figures and Tables

**Table 1:** Some of the commonly employed nutraceuticals in the management of depression as an adjunctive therapy, which thereby present a curative approach [82,83].

Compound	Mechanism of Action
**Omega-3 Fatty Acid Molecules**	They act by inhibiting re-uptake of monoamines during neurological transmission and benefits neurotransmission by increasing the fluidity in membranes of cell. These molecules decrease inflammatory mediators and their synthesis, enhancing neurogenesis and prevents depressive episodes [85].
**N-acetyl Cysteine**	It mainly comprises of anti-inflammatory and antioxidant activities which leads to replenishment of glutathione levels and enhances neurogenesis. It also protects the individual against mitochondrial toxicity and modulates glutamate pathway thereby preventing depression.
**S-adenosyl Methionine**	It mainly influences the production and biotransformation of neurotransmitters as it is an important methyl donor of methyl groups. It also decreases the secretion of prolactin and increases the conversion of phosphatidylcholine [86].
**L-Tryptophan/5-HTP**	Tryptophan is required for conversion into serotonin in the presence of B6 and magnesium to actively form 5-HTP through intermediate processes. The augmentation of tryptophan with a range of antidepressants has been found to be effective in increasing effect. It is used in concert with a range of antidepressants, protein deficient, or in patients with dysregulated serotonergic pathways.
**Vitamin D**	Vitamin D is a ‘neurosteroid’ compound that acts as a ligand for receptors that are present in the hypothalamus, substantia nigra and prefrontal cortex region of the brain. It chiefly regulates the genetic expression leading to coding of protein tyrosine hydroxylase.
**Zinc**	Zinc is the most predominant trace element found in the hippocampus, amygdala and neocortex regions of the brain. It mainly leads to amplification of neurogenesis in hippocampal regions by increasing BNDF. The activity of glutamate and NMDA receptors is also modified.

**Table 2:** Summary of the nutraceuticals discussed in the current review with their mode of action and specific disease activity [64,82,83].

Disease	Mechanism of Action and Commonly Used Nutraceuticals
**Neurodegenerative Disorders**	Neurodegenerative disorders are mainly developed by protein misfolding. Nutraceuticals mainly prevent misfolding of proteins by inhibiting the activation and synthesis of proinflammatory cytokines and associated pathways.Example: bacoside A, bacoside B, brahmine, quercetin, kaempferol, withanine, somniferine, asiatic acid, bhilavanol A and B.
**Alzheimer’s Disease**	AD is mainly associated with increase in oxidative stress and free radicals. The nutraceuticals typically antioxidant in nature are mostly employed in the management of this disease. Examples: flavonoids (fruits, vegetables, tea, wine, coffee); carotenoids (lutein, zeaxanthin, lycopene, β-cryptoxanthin including α and β carotenes); anthocyanidins (cyanidin); flavones (luteolin, apigenin).
**Parkinson’s Disease**	The uninhibited oxidative stress and free radicals in association with abnormally misfolded proteins, neuroinflammation, and dysfunctional mitochondria lead to compromised cellular metabolism and energy thereby impacting the functioning of the brain and leading to neurodegenerative disorders including PD.Examples: Vitamin A, Omega-3 fatty acids, lycopene, vincamine, gallic acid, curcumin, Mito Q.
**Depression**	Nutraceuticals that act by inhibiting re-uptake of monoamines, possess anti-inflammatory and antioxidant properties which are well suited for management of depression.Examples: Omega-3 fatty acids, folic acid, S-adenosyl methionine, zinc, N-acetyl cysteine, L-Tryptophan/5-HTP, Vitamin-D.
**Psychosis**	Nutraceuticals that can improve neurotransmission in dopaminergic serotoninergic neurons can be employed in management of psychosis. These mainly includes all types of vitamins and omega 3 fatty acids.
